# Development of a Surgical Data Availability and Transferability Assessment Tool and Its Use in Evaluating Mozambique's Surgical Data Ecosystem

**DOI:** 10.1002/wjs.70507

**Published:** 2026-08-05

**Authors:** Isménia Simbine, Greta L. Davis, Emilia V. Noormahomed, Chadreque Muluana, Edson Francisco, Stephen W. Bickler, Matchecane Cossa, John Rose

**Affiliations:** ^1^ Surgery Department Maputo Central Hospital Eduardo Mondlane University Maputo Mozambique; ^2^ Center for Health Equity in Surgery and Anesthesia University of California San Francisco San Francisco California USA; ^3^ Division of Plastic and Reconstructive Surgery Department of Surgery University of California San Francisco Medical Center San Francisco California USA; ^4^ Department of Microbiology Faculty of Medicine Universidade Eduardo Mondlane (UEM) Maputo Mozambique; ^5^ Ministry of Health (MISAU) Maputo Mozambique; ^6^ Department of Surgery Division of Pediatric Surgery University of California San Diego La Jolla California USA; ^7^ Rady Children's Hospital San Diego California USA; ^8^ Philip R. Lee Institute for Health Policy Studies University of California San Francisco Medical Center San Francisco California USA

**Keywords:** data ecosystem assessment, health information systems, surgical metrics, surgical systems strengthening

## Abstract

**Background:**

In 2015, the Lancet Commission on Global Surgery promoted the adoption of six perioperative care indicators and increased their visibility in national health policies. However, regular reporting of these surgical indicators remains limited due to challenges with data infrastructure and workforce capacity. This study defines the surgical data ecosystem in a low‐income country and develops a tool for harmonizing national data ecosystems with validated global indicators.

**Methods:**

This exploratory, mixed‐methods study examined the scope of Mozambique's surgical data ecosystem in 2023. Data were obtained in three phases: qualitative interviews with health data stakeholders, analysis of data instruments from four purposively selected public hospitals, and review of aggregate Ministry of Health reports used for surgical system monitoring and evaluation. These data were organized by domains and compared with international reporting standards, informing development of the Surgical Data Availability and Transferability Assessment (S‐DATA) tool for evaluating surgical data availability, transferability, and usability for global benchmarking.

**Results:**

Metadata and data instruments were inconsistent across hospitals, which carried into aggregate reports. Facility‐level data elements and national reports did not align with national and international guidelines, respectively. Processes for aggregating data varied between data managers, leading to loss of information in summary reports. The flow of surgical data from facility to national reports revealed multiple parallel data streams. These findings informed development and pilot application of the S‐DATA tool, with variable data ecosystem performance at the facility level, ranging from fair to poor, yet excellent metadata availability at the highest levels of aggregation.

**Conclusions:**

This study represents the first structured evaluation of a surgical data ecosystem in Mozambique and the development of a novel framework for benchmarking surgical data availability against international standards. Data management analysis revealed gaps, redundancies, and inefficiencies at each level of aggregation. Standardized data instruments and reporting processes may facilitate monitoring and evaluation of surgical systems. Future studies are necessary to validate the S‐DATA tool in additional settings.

## Graphical abstract

In 2015, the Lancet Commission on Global Surgery promoted the adoption of six perioperative care indicators and increased their visibility in national health policies. However, regular reporting of these surgical indicators is limited due to challenges with data infrastructure and workforce capacity. This study defines the surgical data ecosystem in a low‐income country and develops a tool for harmonizing national data ecosystems with validated global indicators. This study represents the first structured evaluation of a surgical data ecosystem in Mozambique and the development of a novel framework for benchmarking surgical data availability against international standards. Data management analysis revealed gaps, redundancies, and inefficiencies at each level of aggregation. Standardized data instruments and reporting processes may facilitate monitoring and evaluation of surgical systems. Future studies are necessary to validate the S‐DATA tool in additional settings.

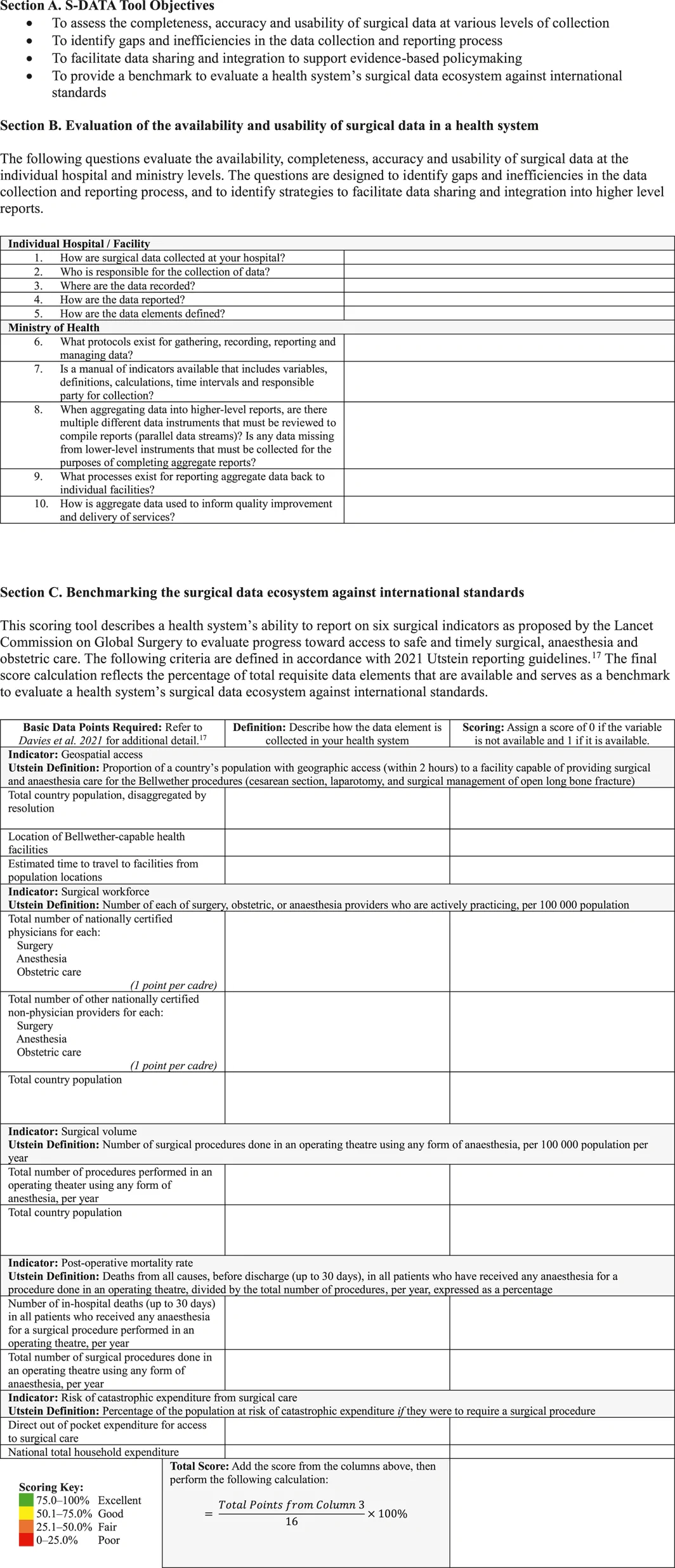

## 1 Introduction

Surgery plays an essential role in sustainable health systems. However, it gained visibility in global health policy only recently after the passage of World Health Organization (WHO) Resolution 68/15, which called for regular and reliable measures of access to surgical care worldwide [1]. In response, the Lancet Commission on Global Surgery (LCoGS) proposed a suite of indicators for monitoring and evaluating surgical services with global targets for 2030 [2]. According to recent estimates, nearly half of all countries had active or expired national surgical, obstetric, and anesthesia plans (NSOAPs) or provisions for surgery within national health plans; despite this, routine reporting of surgical indicators remains inconsistent [3, 4]. The latest estimates reveal slow progress toward Global Surgery 2030 targets: only 26% of low‐ and middle‐income countries (LMICs) are on track to meet access targets, 41% for workforce targets, and 0% for surgical volume targets [4].

Despite the recent increase in national surgical indicator reports, few countries have achieved routine reporting due to challenges with data infrastructure, collection, and management [5, 6]. Past efforts have been defined by one‐time research exercises that failed to integrate within national health information systems and, therefore, did not support sustainable reporting [3, 7, 8, 9, 10, 11]. There is an urgent need to embed surgical metrics within national health data architecture to facilitate ongoing monitoring and evaluation.

Mozambique is one of the world's poorest nations with a total population of 33.6 million and carries a high burden of morbidity and mortality for surgically treatable conditions due to limited access, inadequate workforce density, and insufficient infrastructure [12, 13, 14]. In 2015, a nationwide surgical dataset was compiled to benchmark performance against LCoGS targets [14], yet the current ecosystem lacks capacity for routine, timely, and needs‐responsive surgical data collection [15]. This carries important implications for health outcomes, as surgical care delivery is impacted by surgical delays, inefficient resource allocation, and limited policy responsiveness [16]. Despite the centrality of data to surgical system strengthening, no framework exists to systematically assess national surgical data ecosystems. This study aims to define the collection, storage, sharing, and dissemination of surgical data in Mozambique and to introduce a framework for evaluating and benchmarking a national surgical data ecosystem against internationally validated indicators.

## 2 Material and Methods

This exploratory, mixed‐methods study examined the scope of Mozambique's surgical data ecosystem and is reported in accordance with the Consolidated Criteria for Reporting Qualitative Research (COREQ). The study was approved by the Institutional Review Board (IRB) of the Mozambique Ministry of Health (MOH).

Data on Mozambique's surgical informatics were obtained via semistructured interviews with stakeholders in health data management at the facility, district, and national levels. Participating facilities were purposively selected from Maputo City and Maputo Province based on feasibility of site visits, geographic accessibility, and the presence of at least one functional operating suite. Seven public hospitals with operating suites were located within the study region, and four were purposively selected for this pilot evaluation. All four facilities agreed to participate.

Semistructured interviews explored how and which surgical data and metadata are captured, aggregated, and transferred from facility‐level reports to district and national summary reports. Data instruments and aggregation protocols were compared across sites and against international reporting templates [2, 17]. The roles and responsibilities of stakeholders involved in health data management across reporting levels were defined.

### 2.1 Interview Procedure and Research Team Characteristics

Interviews were conducted by two trained researchers (one male and one female physician‐researcher with prior qualitative research training and backgrounds in system‐level surgical planning). There were preexisting professional relationships between the research team and some MOH officials due to clinical work and prior collaborations, which were disclosed to participants. No financial incentives were provided. Participants verbally consented to in‐person interviews. Detailed field notes were taken during and immediately after each interview, and findings were presented during a stakeholder symposium for validation and discussion. All healthcare data were presented as summary reports and contained no identifiable patient information.

### 2.2 Stakeholder Analysis

Stakeholders were mapped to a salience stakeholder framework to define engagement strategies and consider differing positions and interests [18]. Entities that influence or are influenced by the management, delivery, and improvement of the national health system, particularly concerning surgical care, and who agreed to participate, were considered as stakeholders for the purpose of this study. Stakeholder groups included policymakers, data and analytical professionals, hospital administrators, and clinical providers to ensure that the “optimal” data ecosystem reflected of the diverse needs of all interested parties. Stakeholder definitions, roles, and interests are defined in Box 1.

**BOX 1 Stakeholder definition by category and vested interest.**

1

**Table:** 

Stakeholder Group	Definition	Role and Vested Interest
Policymakers	Representatives from the MOH who are responsible for overseeing and implementing policies related to surgical care and health system management	Interested in data systems that support effective policy implementation and resource allocation
Data and analytical professionals	Statisticians and data scientists who handle the collection, analysis and interpretation of health data	Require accurate and comprehensive data for analysis to inform evidence‐based decision‐making and health policy
Hospital administrators	Administrators who manage day‐to‐day health facility operations, including the integration of surgical data systems into operational workflows	Rely on integrated data systems for operational efficiency, resource management, and quality improvement
Surgical providers and teams	Surgeons and perioperative team members who are directly involved in patient care	Attune to how data impacts clinical practice, quality improvement, resource availability and operational processes in surgical care delivery

### 2.3 Surgical Indicator Symposium

An in‐person, 2‐day stakeholder symposium was organized to review and validate findings regarding facility‐level metadata variability. Additional data instruments and national reporting guidelines were discovered and compared against international, aggregate reporting templates from LCoGS and the Utstein Surgical Metrics Group [2, 17]. Breakout groups focused on aligning national data reporting mechanisms with international standards, eliminating redundancies, and addressing missing metadata. Consensus on proposed improvements was achieved through plenary discussion, and recommendations were consolidated into a set of amendments to the national Manual of Indicators and presented to MOH leadership for approval.

### 2.4 Development of the Surgical Data Availability and Transferability Assessment (S‐DATA) Tool

The scientific and grey literature were referenced to compile an exhaustive list of domains and subdomains related to health data ecosystems, which were further modified for relevance to surgical systems (Supporting Information S1: Appendix 1). This list served as a framework to understand the availability, completeness, accuracy, and usability of surgical data at successive levels of aggregation. Key references included international surgical indicator definitions and broader health system assessment tools from the LCoGS, the Utstein Surgical Metrics Group, the United Nations, the World Bank, and the WHO [17, 19, 20, 21, 22, 23, 24, 25].

Referencing these domains and lessons learned during Mozambican hospital assessments, we developed the Surgical Data Availability and Transferability Assessment (S‐DATA) Tool, the principal methodological output of this study (Figure 1). Six domains were evaluated: information management, financing, human resources, governance, medical technology and infrastructure, and service delivery. The primary objectives of the tool were fourfold: (i) to assess the completeness, accuracy, and usability of surgical data at various levels of collection; (ii) to identify reporting gaps and inefficiencies; (iii) to facilitate data sharing and integration for evidence‐based policymaking; and (iv) to establish a benchmark against international surgical reporting standards according to the proportion of requisite data elements available [17]. Performance was defined as “excellent” (≥ 75.1% of surgical data elements present), “good” (50.1%–75.0%), “fair” (25.1%–50.0%), and “poor” (≤ 25.0%). The resulting framework was piloted across the four healthcare facilities to evaluate its clarity, feasibility, and applicability, and iterative refinements were made to improve the tool prior to its presentation in Figure 1.

FIGURE 1Surgical Data Availability and Transferability Assessment (S‐DATA) Tool. Figure 1 is the S‐DATA tool structure which includes three sections: Section A (objectives, bullet points), Section B (evaluation questions for individual hospital and Ministry of Health levels in tabular format), and Section C (benchmarking framework with indicators, definitions, and scoring). Tables include domains such as data collection processes, metadata availability, aggregation, and use of data, and scoring criteria based on availability of required data elements for global surgical indicators adapted from Davies et al. 2021 [19].

**Figure:**
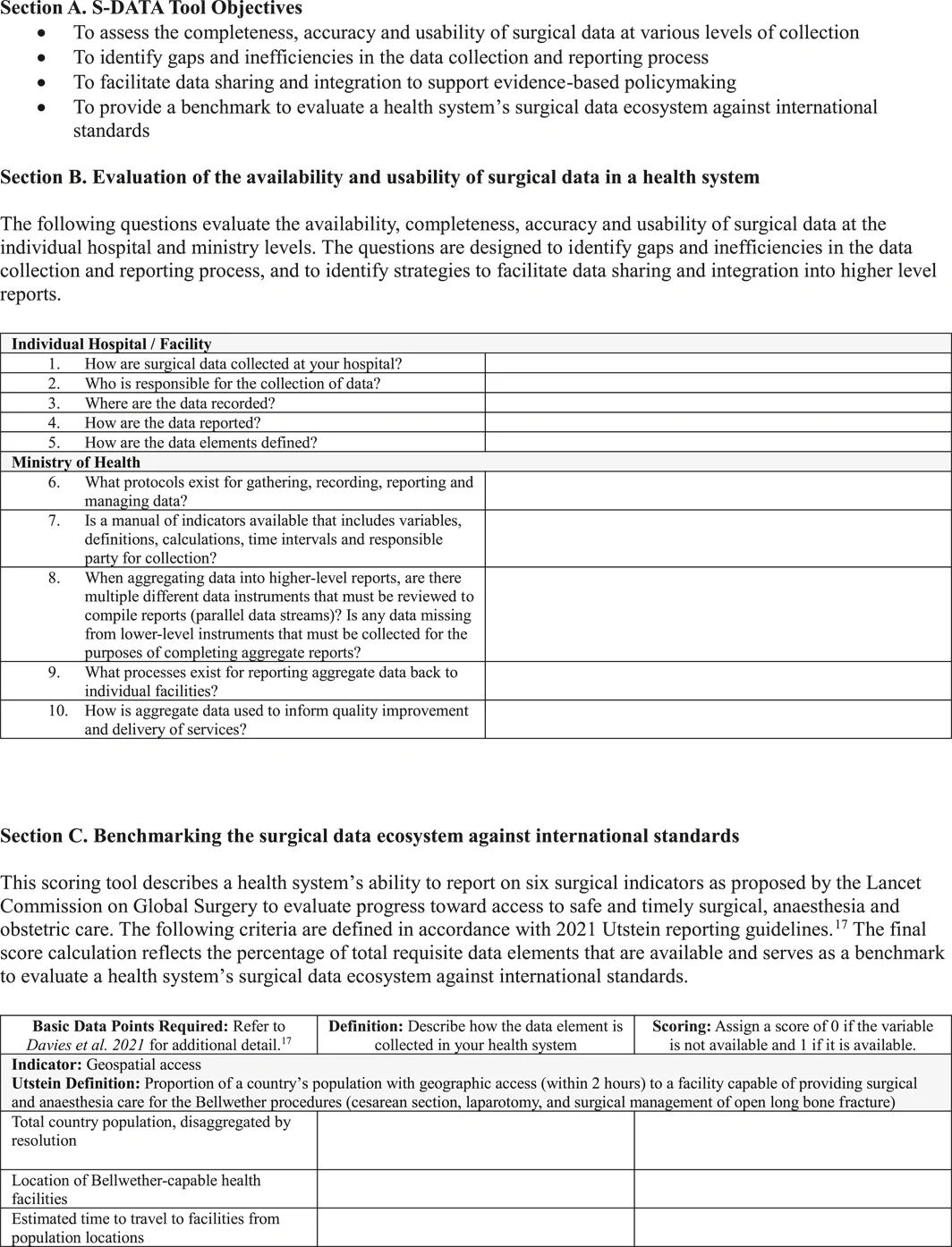

**Figure:**
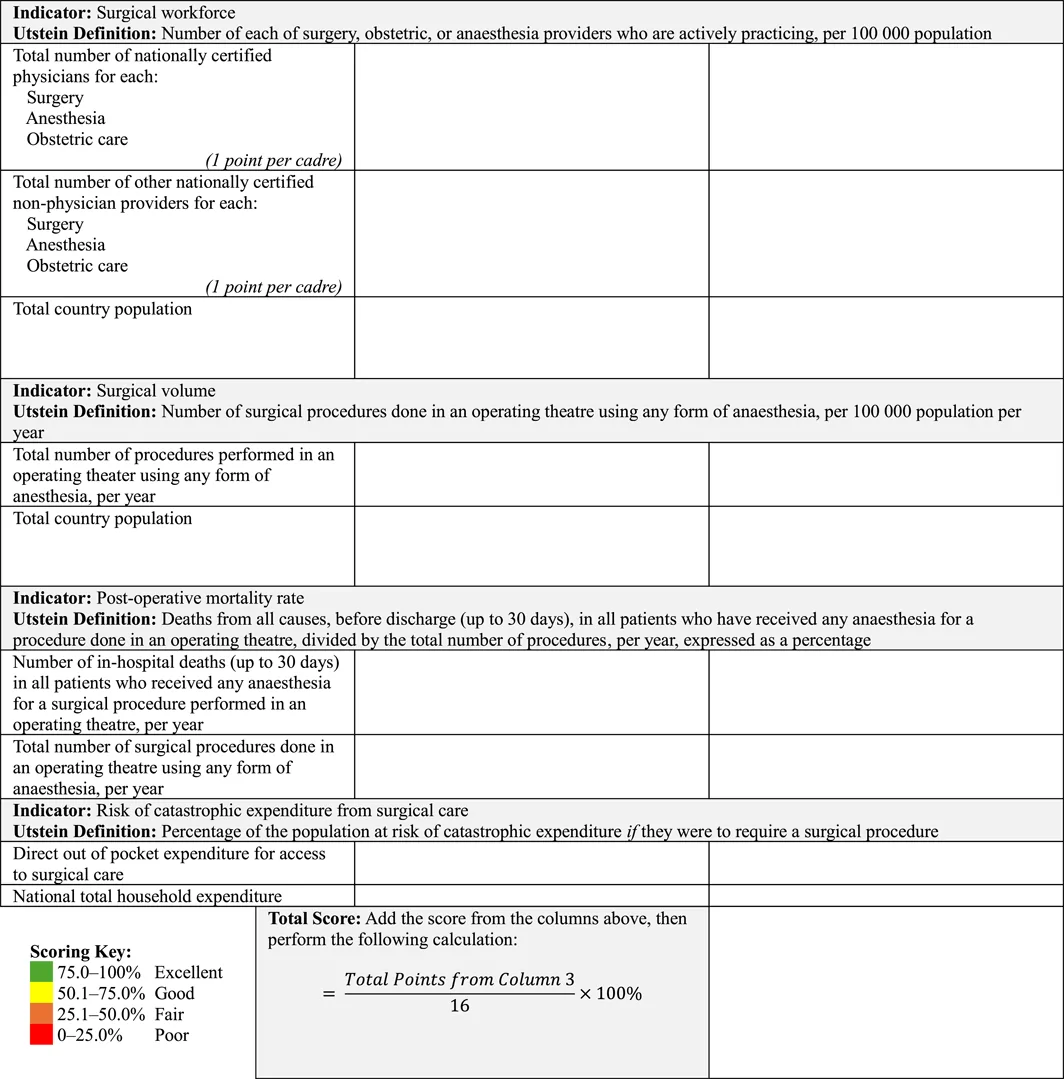

## 3 Results

### 3.1 Overview of Mozambique's Surgical Data Ecosystem

**FIGURE 2:**
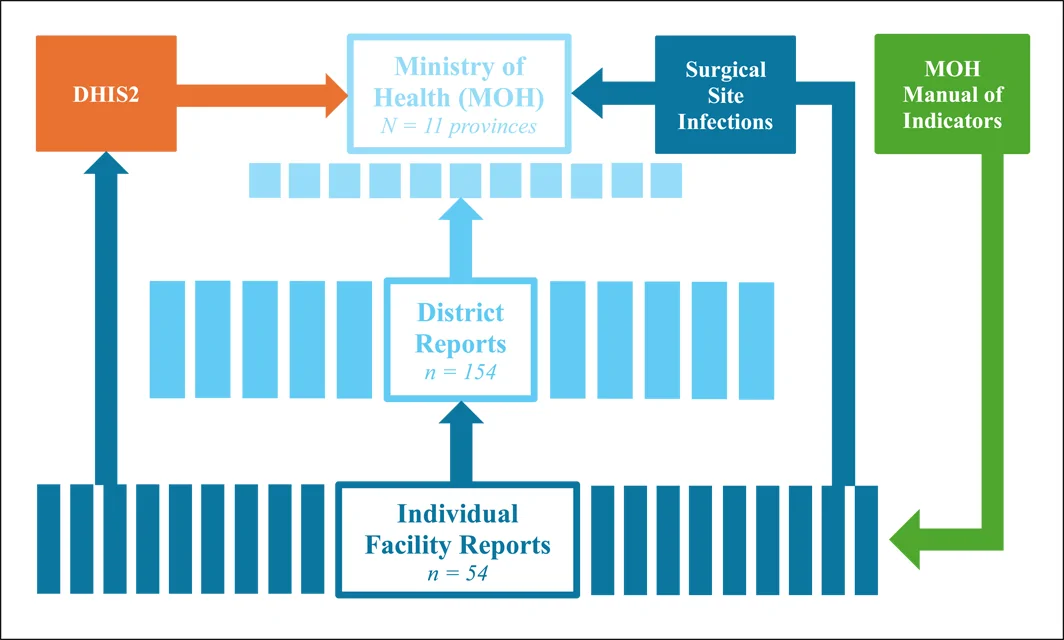
Flow of surgical data within the Mozambican health system. Figure 2 is a diagram showing surgical data flow across facility (Individual Facility Reports, *n* = 54), district (District Reports, *n* = 154), and national (Ministry of Health, *n* = 11 provinces) levels. Rectangular boxes represent reporting units, solid arrows indicate primary data flow from facility to district to national reporting units and national reports, and additional arrows depict parallel streams including DHIS2 modules, surgical site infection reporting, and the MOH Manual of Indicators. Horizontal dashed bands denote levels of aggregation.

The flow of surgical data within the Mozambican health system was defined (Figure 2). In most cases, individual facilities generated monthly reports, which were aggregated at the district level and then summarized in annual MOH reports. This process was informed by an MOH Manual of Indicators, which outlined metadata and definitions for nine surgical delivery and quality indicators. Several parallel data streams were identified: (i) a discrete process for reporting surgical site infections to the MOH; (ii) multiple surgical modules hosted by the DHIS2 platform, which collects surgical volume and postoperative mortality for a select list of procedures in general surgery and ophthalmology; and (iii) provincial reports, which bypass the aforementioned facility‐to‐district aggregation exercise.

### 3.2 Evaluating the Volume and Consistency of Metadata

Next, four purposively selected hospitals from Maputo City and Maputo Province were approached and all agreed to participate in site assessments: Mavalane General Hospital (MGH), José Macamo General Hospital (JMGH), Manhiça District Hospital (MDH), and Xinvane Rural Hospital (XRH). All hospitals were public, secondary care facilities with varied operative capacity (1–4 functioning operating rooms), size (53–383 inpatient beds), and annual population served (44,223–409,835) (Table 1). Data reporting at each facility was managed by surveillance technicians who utilized distinct data instruments by location: operative room/recovery area, intensive care unit, and surgical ward. All data were reported on paper forms except for MGH, which utilized an electronic medical record. Data from each location were manually entered into an electronic system every month and then aggregated into district reports at the same frequency. These district reports were submitted to the MOH.

**TABLE 1:** Facility‐level variation in surgical data instruments and metadata across hospitals.

	Mavalane general hospital	José Macamo general hospital	Manhiça district hospital	Xinvane rural hospital
Facility data				
Type	Public	Public	Public	Public
Level	Secondary	Secondary	Secondary	Secondary
Beds	383	335	148	53
Functioning ORs	4	4	1	1
Population served (year)	382,023	409,835	276,211	44,223
Data category	Quantity of metadata^a^			
Population				
Direct catchment	1	1	1	1
Volume of surgery				
Total surgeries	1	1	1	1
By procedure	75	8	8	13
By urgency	2	0	2	2
Total admissions	6	1	1	0
Total discharges	1	1	1	0
Mortality, inpatient				
Total	1	1	1	1
By procedure	8	8	8	12
Workforce				
Surgeons	1	1	1	1
Surgical technicians	1	1	1	1
Anesthesiologist	1	1	1	1
Anesthesia technicians	1	1	1	1
Obstetricians	1	1	1	1
Infrastructure				
Beds, surgical ward	1	1	1	1
Operating rooms				
Total	0	0	0	0
Functional	1	1	1	1
Anesthesia machines				
Total	1	1	1	1
Functional	0	0	0	0
Disposable equipment	0	0	0	5
Quality improvement				
Cancellations	0	1	0	0
Readmissions	0	1	0	0
M&M discussions	1	1	1	0

Abbreviation: M&M = morbidity and mortality conference.

^a^ See Table S1 for detailed list of variables and metadata collected.

Daily logbooks and periodic summary reports were inspected at each facility, and the total number of variables collected by category were tabulated (e.g., catchment population, workforce, infrastructure, volume, mortality, quality improvement) (Table 1). There was significant variability in the quantity of metadata collected per category. For example, surgical volume was reported for 75 distinct procedures (33 elective, 31 urgent, and 11 unspecified) by MGH, whereas JMGH and MDH reported volume for notably fewer procedure types (8 items). Instead, JMGH applied greater detail in its reporting of surgical discharges by diagnosis (23 items). All hospitals reported inpatient mortality, with varied level of detail according to procedure type. There was inconsistent reporting on infrastructure and quality improvement items, such as surgical cancellations, readmissions, and morbidity along with mortality discussions. A comprehensive list of variables collected at each facility and by level of aggregation is presented in Table S1.

Aggregate reports were less variable due to the use of standardized data instruments. For example, the DHIS2 instrument was used by all four facilities and consistently collected total surgical volume, total surgical discharges, and total inpatient surgical deaths for eight predefined procedures (elective and emergent inguinal hernia repair, laparotomy, elective and emergent cesarean delivery, hysterectomy, and tubal ligation) and an “other” category. Notably, treatment of open fractures, one of the three Bellwether procedures, was not captured at this level.

MOH Annual Reports were available for review at two of the four facilities and contained predefined data categories yet variable reporting of items within each category. For example, surgical volume and postoperative mortality were reported only for the five most common procedures and “other” performed at each facility, which introduced variability. Although the bellwether procedures likely fall into the five most common procedures for general, obstetric, and orthopedic surgery, these were not explicitly included. Metadata relevant to surgical care, including catchment population, perioperative workforce figures, and hospital and operative bed capacity, were available from nonclinical reports. Interestingly, 92.0% of the inpatient clinical metadata required to complete the MOH Annual Report were not gathered by the DHIS2 instrument (e.g., admissions, procedures performed, and mortality rate for the top 5 indications for general, obstetric, and orthopedic surgery) and 66.0%–94.0% were absent from facility reports, suggesting the presence of additional, parallel data streams to obtain the necessary data. The quantity of metadata collected from facilities to broader summary reports is detailed Table S2.

### 3.3 Surgical Data Availability and Transferability Assessment (S‐DATA) Tool

The principal product of this study is the S‐DATA tool (Figure 1), a structured framework developed from these findings to evaluate the availability, transferability, and reportability of surgical data while benchmarking individual facilities and their broader health systems against internationally recognized surgical indicators.

Each of the four participating facilities was evaluated using the S‐DATA tool (Table 2), which revealed fair facility‐level metadata availability at MGH (41.7%) and XRH (41.7%), and poor metadata availability at JMGH (25.0%) and MDH (16.7%). However, when considering data at all levels of aggregation, including DHIS2 and annual MOH reports, 10 of the 12 requisite metadata were available from all four hospitals (83.3%; excellent). Table 3 presents an overview of metadata availability by instrument and aggregation level across the health system. These findings suggested the need for a common set of variables to collect at the facility, district, and national levels to standardize reporting and eliminate parallel data streams.

**TABLE 2:** Application of the S‐DATA tool to assess surgical data availability across four hospitals.

	Facility			
	MGH	JMGH	MDH	XRH
Indicator 1: Geospatial access				
Total population	Yes	Yes	Yes	Yes
Bellwether capability	Yes^a^	No	No	Yes^a^
Estimated time to travel to facility	No	No	No	No
Indicator 2: Surgical workforce				
Total surgeons	No	No	No	No
Total nonphysician surgeons	No	No	No	No
Total anesthesiologists	No	No	No	No
Total nonphysician anesthesia providers	No	No	No	No
Total obstetricians	No	No	No	No
Total nonphysician obstetric providers (“maternal and child health technician”)	N/A^b^	N/A^b^	N/A^b^	N/A^b^
Indicator 3: Surgical volume				
Total number of procedures	Yes	No	No	Yes
Total population	Yes	Yes	Yes	Yes
Indicator 4: Postoperative mortality rate				
Total inpatient deaths	No	Yes	No	No
Total number of procedures	Yes	No	No	Yes
Indicator 5: Risk of catastrophic expenditure				
OOP Expenditure for surgery	N/A^c^	N/A^c^	N/A^c^	N/A^c^
National household expenditure	N/A	N/A	N/A	N/A
Facility score^d^				
Scoring key				
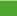 75.0%–100% excellent	41.7%	25.0%	16.7%	41.7%
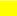 50.1‐75.0% good				
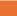 25.1‐50.0% fair				
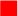 0–25.0% poor				

Abbreviations: JMGH = José Macamo General Hospital, MDH = Manhiça District Hospital, MGH = Mavalane General Hospital, OOP = out of pocket, XRH = Xinvane Rural Hospital.

^a^ Item is not directly reported, but ascertainable from available metadata.

^b^ Provider category does not exist in the Mozambican health system and is therefore not considered against total metadata requirements.

^c^ Not applicable as patients do not pay out of pocket for insurance or healthcare services and is not considered against total metadata requirements.

^d^ Score calculated as detailed in S‐DATA tool: (total metadata listed as available/12) * 100%. Note that of the 16 listed categories of metadata, only 12 are relevant to the Mozambican health system and therefore a denominator of 12 is used for this calculation.

**TABLE 3:** Surgical data instruments and metadata availability across levels of health system aggregation.

Level of aggregation	Data instrument (frequency reported)	Quantity of metadata by category^a^
Multinational	Global summary reports (variable) *Lancet Surgical Indicators*	I. Service delivery a. Workforce (7) II. Access a. Geospatial access (2) b. Financial risk (0) III. Quality and safety a. Volume (1–2, depending on facility) b. Mortality (1–2, depending on facility)
National	National summary reports *MOH Annual Report (annual)* ^b^	I. Service delivery a. Disease burden (10) b. Workforce (5) c. Infrastructure (11) II. Access a. Population (2) b. Bellwether facilities (0) III. Quality and safety a. Volume (20) b. Mortality (20) c. Quality improvement (4)
	*DHIS2 (monthly)*	I. Service delivery a. Disease burden (0) II. Quality and safety a. Volume (2) b. Mortality (9)
	Manual of indicators (one‐time)	I. Service delivery a. Disease burden (0) b. Workforce (0) c. Infrastructure (1) II. Access a. Population (0) b. Bellwether facilities (0) III. Quality and safety a. Volume (5) b. Mortality (2) c. Quality improvement (5)
	Population reports (decennial) *Population and Housing Census*	I. Service delivery a. Population i. Total (1) ii. Gender (2) iii. Urban‐rural classification (2)
Facility	Individual patient record (daily) *Paper chart/electronic health record* *Ward/unit logbook* *Operative logbook*	I. Service delivery a. Workforce: 5 (range across facilities) b. Infrastructure: 3–8 (range across facilities) II. Access a. Catchment population: 1 b. Bellwether capability: 0 III. Quality and safety a. Volume: 11–85 (range across facilities) b. Mortality: 9–13 (range across facilities) c. Quality improvement: 0–3 (range across facilities)

Abbreviation: DHIS2 = District Health and Information Software 2, an open‐source platform for health management data (https://dhis2.org/about‐2/#:~:text=DHIS2%20is%20an%20open%20source,management%20information%20system%20(HMIS), MOH = Ministry of Health.

^a^ See Table S2 for detailed list of variables and metadata collected.

^b^ The Manual of Indicators is a one‐time MOH publication derived from a consensus process that includes definitions, calculations, and time intervals for reporting of health‐related variables from individual facilities. Ministério da Saúde. Direcção Nacional de Assistência Médica. Departamentos de Administração e Gestão das Unidades Sanitárias. “Manual de indicadores de desempenho de hospitalar”. Moçambique: MISAU/DNAM/DAGUS. 2023.

## 4 Discussion

This study represents the first endeavor to evaluate the surgical data ecosystem in Mozambique using a multistakeholder approach and, in doing so, develops the S‐DATA tool as a structured framework for evaluating and benchmarking surgical data ecosystems. The primary finding from this analysis is that surgical data collection relative to global standards is inadequate and that multiple, parallel data streams exist due to a lack of centralized processes for data reporting and management.

Hospital site visits and data instrument evaluation revealed significant variability in the volume, format, and type of available metadata. Although no studies have performed a comprehensive assessment of facility‐level surgical data reporting, heterogeneity in surgical indicator definitions is a common finding [6, 9]. This heterogeneity hinders the quality and usefulness of data gathered from routine reports, including patient medical records and facility logbooks, which remain the primary data source in existing publications on surgical indicator availability and performance [7, 9, 10, 11]. Mahmood and colleagues performed a similar analysis of Pakistan's health data ecosystem and identified large amounts of available data but limited capacity for its utilization in policy planning and decision‐making [26]. Poor coordination between clinicians, researchers, and policymakers contributes to a lack of consistency and conciseness of available health data. Thus, an important early step is engaging multisectoral stakeholders from clinical, research, policy, and other backgrounds to define a “minimum essential package” of facility‐level surgical indicators and metadata to improve data validity and usefulness while still allowing for contextual modification through the addition of facility‐specific metrics. Immediate needs for strengthening Mozambique's surgical data ecosystem include increasing the quantity, quality, and consistency of metadata collected. While studies that measure the benefits of data utilization in low‐resource settings are limited [27], prior publications in high‐income settings demonstrate that efforts to improve health information access and usability can produce notable economic benefits amounting to $1.2 to $5 billion USD [28].

This study noted few similarities between ward logs and aggregate reporting templates, which represented a barrier to streamlined, higher‐level reporting. Variability decreased at successive levels of aggregation due to the use of standardized data instruments such as the DHIS2 module and MOH Annual Report, which were accompanied by a Manual of Indicators with well‐defined inputs. However, residual inconsistencies between reports suggest a need for improved standardization and integration across all levels of aggregation. Parallel data streams have been identified in nonsurgical data systems and contribute to an increased burden of data collection, inconsistent documentation, and poor interoperability [29, 30, 31, 32, 33]. Although redundancies and inefficiencies have been reported across country income levels [28, 34, 35], the implications are more severe for countries that carry a disproportionate healthcare burden [36]. Moving forward, clearly defined and standardized data instruments are needed to ensure that data and metadata are readily available for summary reports and integrate well into existing infrastructure.

Despite these challenges, there was a widespread interest from stakeholders in establishing a surgical data ecosystem with appropriate governance, intentionality of data design elements, and inclusion of metadata that is both needs‐responsive and patient‐centered. Multistakeholder engagement represents a critical strategy for value co‐creation by defining the inputs, activities, and desired outputs that guide movement from the current to the ideal surgical data ecosystem [37, 38]. This approach has proven to be highly effective and feasible in NSOAP processes, which have enabled increased prioritization of surgery in national health agendas [39]. However, such processes often rely on expert opinion and stakeholder input rather than robust empirical evidence [40, 41]. The findings from this study can inform strategies to strengthen surgical data ecosystems that are capable of producing reliable and timely evidence to guide national surgical planning and support routine monitoring and evaluation.

In the business sector, methodologies for evaluating complex data ecosystems are well established, with numerous assessment frameworks available to guide implementation and performance measurement [42]. Although the WHO's SCORE (Survey, Count, Optimize, Review, Enable) package provides similar benchmarking and scoring frameworks for health information systems [43], no surgery‐specific tool exists. The S‐DATA tool uniquely focuses on surgical care and defines surgical indicator reportability by available metadata within existing reporting structures.

The S‐DATA tool revealed significant metadata variability between facilities and limited capacity to generate comprehensive surgical indicator reports. Although surgical volume and postoperative mortality were generally reported, it was impossible to disaggregate by procedure due to missing or inconsistent procedural classification systems. In the MOH Annual Reports, the practice of collecting volume and mortality data only for the five most common procedures introduced selection bias and interfacility variability. The S‐DATA tool also revealed inconsistent workforce and catchment reporting and no financial risk protection data. Mozambique's National Health Service mandates provision of healthcare to the entire population despite persistent challenges related to governance, service quality, and resource mobilization; current definitions of financial risk, which rely on measuring out‐of‐pocket payments for direct medical costs, are difficult to operationalize in this care provision model and may explain the absence of reporting on this indicator [17]. Nonetheless, the observed variability in data quality and availability across facilities further supports the need to establish a more robust surgical data ecosystem.

This study has several limitations. Although broad multidisciplinary perspectives were represented in stakeholder analysis, patient and community perspectives were not captured. These viewpoints are essential for understanding how data systems translate into service delivery and patient outcomes. Facility‐level assessments were intentionally limited to a purposive sample of public secondary‐care hospitals in Maputo City and Maputo Province because of feasibility, travel, and security considerations, which precluded broader national site visits during this pilot evaluation. Although all participating facilities represented diverse operating capacity, size, and catchment populations, the sample was not intended to be nationally representative. Thus, findings should be interpreted as an in‐depth evaluation of the surgical data ecosystem within the study setting rather than at the national level. Data instruments were assessed at a single point in time, and conclusions regarding metadata availability assume that facilities submit complete and accurate reports at the required frequency. Nonetheless, this study provides a novel tool to evaluate surgical data ecosystem performance in low resource settings.

Future directions include evaluating the S‐DATA tool nationwide before broader validation and developing standardized data collection and reporting protocols to enable monitoring of surgical care delivery in Mozambique. This is important because prior work demonstrated widespread deficiencies in surgical care outside the capital of Maputo [14]. A robust data ecosystem capable of monitoring surgical services will be an important step toward identifying areas of greatest need, informing ongoing national surgical planning and increasing investment in surgical care.

## Author Contributions

**Isménia Simbine:** data curation, formal analysis, investigation, methodology, project administration, writing – original draft, writing – review and editing. **Greta L. Davis:** data curation, formal analysis, investigation, methodology, project administration, writing – original draft, writing – review and editing. **Emilia V. Noormahomed:** validation, writing – review and editing. **Chadreque Muluana:** data curation, methodology, validation, visualization. **Edson Francisco:** data curation, methodology, validation, visualization. **Stephen W. Bickler:** validation, writing – review and editing. **Matchecane Cossa:** conceptualization, data curation, investigation, supervision, validation, writing – review and editing. **John Rose:** conceptualization, data curation, investigation, supervision, validation, writing – review and editing.

## Funding

University of California Perioperative and Surgical Health Equity Center Seed Award. The study sponsor was not involved in the study design; collection, analysis, or interpretation of data; in the writing of the report; or in the decision to submit this paper for publication.

## Ethics Statement

The study was approved by the Institutional Review Board (IRB) of the Ministry of Health of Mozambique.

## Consent

The authors have nothing to report.

## Conflicts of Interest

The authors declare no conflicts of interest.

## Supporting information


Supporting Information S1

**Table 1:** Surgical data instruments and variables collected at the facility level.

**Table 2:** Surgical data instruments and variables collected at the national level.

## Data Availability

The data that supports the findings of this study are available in the supplementary material of this article.
