# Supplementary material for: Development of a Surgical Data Availability and Transferability Assessment Tool and Its Use in Evaluating Mozambique's Surgical Data Ecosystem
Source: World J Surg. 2026 Aug 5;50(9):2680–91. doi: 10.1002/wjs.70507 (PMC13576621; doi:10.1002/wjs.70507)
Supplement: Supplementary file 1 [file WJS-50-2680-s001.docx]

**Supplemental Table 2. Surgical data instruments and variables collected at the national level**

|  | **Data Instrument (Frequency Reported)** | | |
| --- | --- | --- | --- |
|  | **Ministry of Health Manual of Indicators (Once)^a^** | **Ministry of Health Annual Report (Annual)** | **Population and Housing Census (Decennial)** |
| **Data Category** | **Variables Collected** | | |
| Service delivery |  |  |  |
| *Disease burden* |  | Total number of primary surgical consultations  Total number of secondary surgical consultations  Most frequent diagnoses leading to admission to surgical ward (5 categories plus ‘other’)  Total admissions to surgical ward  Total discharges from surgical ward |  |
| *Workforce* |  | Total number of surgeons  Total number of surgical technicians  Total number of anesthesiologists  Total number of anesthesia technicians  Total number of obstetricians |  |
| *Infrastructure* | Number of functional operating suites | Number of hospital beds  Number of hospital beds on surgical ward  Number of operating tables  Number of functioning operating tables  Number of anesthesia machines  Number of functioning anesthesia machines  Equipment availability (gloves, syringe, blades, suture, other) |  |
| Access |  |  |  |
| *Population, total* |  | National population  Direct catchment population | National population  By sex (male / female)  By province (11 provinces)  By urban-rural classification (2 categories) |
| Quality & safety |  |  |  |
| *Volume* | Number of emergency surgical interventions  Number of elective surgical interventions  Total number of surgical interventions performed in an operating suite  Number of elective cesarean sections  Total number of cesarean sections | Total number of major surgical operations  Total number of cesarean sections  Most frequent diagnoses leading to emergency surgery (5 categories plus ‘other’)  Total number of surgeries  Most frequent diagnoses leading to emergency obstetric surgery (5 categories plus ‘other’)  Total number of surgeries  Most frequent diagnoses leading to emergency orthopedic surgery (5 categories plus ‘other’)  Total number of surgeries |  |
| *Mortality* | Total number of inpatient deaths on surgical service  Total number of deaths in operating or recovery room | Total number of inpatient deaths on surgical service  Total number of surgical deaths by procedure   - Due to 5 most frequent diagnoses plus ‘other’ leading to emergency surgery - Due to 5 most frequent diagnoses plus ‘other’ leading to emergency obstetric surgery - Due to 5 most frequent diagnoses plus ‘other’ leading to emergency orthopedic surgery   Total number of deaths in operating room or recovery room |  |
| *Quality improvement* | Total number of inpatient deaths on surgical service discussed during morbidity & mortality-equivalent conference  Number of patients with surgical site infection of a clean surgical site  Number of patients undergoing reoperation due to complication from prior surgical intervention  Number of cancelled surgical interventions  Number of scheduled surgical interventions | Total number of inpatient deaths on surgical service discussed during morbidity & mortality-equivalent conference  Total number of avoidable inpatient surgical deaths  Total number of unavoidable inpatient surgical deaths  Number of days waiting for consultation with surgeon |  |

**^a^** The Manual of Indicators is a one-time Ministry of Health publication that informs facilities within the health system on the choice, management, interpretation and use of health indicators and includes the justification, definition, metadata required for calculation, frequency of collection and targets for each listed indicator.
