# Supplementary material for: Development of a Surgical Data Availability and Transferability Assessment Tool and Its Use in Evaluating Mozambique's Surgical Data Ecosystem
Source: World J Surg. 2026 Aug 5;50(9):2680–91. doi: 10.1002/wjs.70507 (PMC13576621; doi:10.1002/wjs.70507)
Supplement: Supplementary file 2 [file WJS-50-2680-s002.docx]

**Supplemental Appendix 1. Comprehensive framework to evaluate data availability within domains of the surgical data ecosystem**

This comprehensive framework provides structure for generating in-depth questions to understand the availability, completeness, accuracy, and usability of surgical data within each domain of the data ecosystem. The selection of domains and sub-domains was primarily informed by key international guidelines on the collection and evaluation of surgical indicators and larger-scope health system assessment tools.^17,19–25^

I. **Governance** (rules, laws and organizational structure required to ensure the integrity, security and effective use of surgical data)
   a. Ethics
   b. Security and privacy
   c. Oversight and accountability
   d. Regulatory / legal
   e. Data use agreement / transparency
   f. Data efficacy
   g. Policies
II. **Financing**
    a. Data acquisition
    b. Database queries
    c. Data storage and maintenance
    d. Data sharing / interoperating
III. **Human Resources / Workforce**
     a. Distribution
     b. Education and training
     c. Readiness / skill badging
     d. Management
IV. **Information Management**
    a. Architecture
    b. Sourcing / sharing
    c. Standards / data dictionary / manual of indicators
       i. Variables
       ii. Definitions
       iii. Calculations
       iv. Metadata
       v. Time intervals
       vi. Responsible party
    d. Quality assurance
       i. Completeness
       ii. Validation / cleaning
       iii. Usability
    e. Analytics and decision-making
    f. Visualization
    g. Modeling
V. **Medical Technology and Infrastructure**
   a. Operations / maintenance
   b. Storage
   c. Display and reporting (e.g. dashboards)
   d. Selection and development
   e. Security
   f. Digitization (incl. location of computers/tablets)
VI. **Service Delivery**
    a. Epidemiology
       i. Census / population data
       ii. Surgical disease burden
    b. Availability
       i. Access
       ii. Financial risk
    c. Capacity / Readiness
       i. Workforce / human resources
       ii. Service distribution
       iii. Facilities / infrastructure
       iv. Equipment / supply chain
       v. Financing
    d. Quality and safety
       i. Volume
       ii. Outcomes
    e. Management
