# Supplementary material for: Development of a Surgical Data Availability and Transferability Assessment Tool and Its Use in Evaluating Mozambique's Surgical Data Ecosystem
Source: World J Surg. 2026 Aug 5;50(9):2680–91. doi: 10.1002/wjs.70507 (PMC13576621; doi:10.1002/wjs.70507)
Supplement: Supplementary file 3 [file WJS-50-2680-s003.docx]

**Supplemental Table 1. Surgical data instruments and variables collected at the facility level**

|  |  | **Data Instrument (Frequency Reported)** | | |
| --- | --- | --- | --- | --- |
|  |  | **Logbook (Daily)** | **DHIS2 (Monthly)** | **Hospital Report (Annual)** |
| **Mavalane General Hospital** | **Data Category** | **Variables Collected** | | |
|  | *Volume* | Total surgeries by surgical procedure, elective *(33 items):*   - Amputation - Appendectomy - Cerclage - Cholecystectomy - Circumcision - Condylectomy - Cystectomy - Debridement, necrotizing fasciitis - Debridement, ulcer - Excision, lipoma - Excision, tumor - Fissurectomy, anal - Fistulectomy, anal - Hemorrhoidectomy - Herniorrhaphy, inguinal - Herniorrhaphy, umbilical - Herniorrhaphy, white line - Hydrocelectomy - Hysterectomy, subtotal - Hysterectomy, total - Incision and drainage, abscess - Incision and drainage, perianal abscess - Laparotomy - Lumpectomy - Mastectomy, benign - Mastectomy, malignant - Myomectomy - Orchiopexy - Scar excision - Skin graft for burn - Thyroidectomy - Varicocelectomy - Other   Total surgeries by surgical procedure, emergent *(31 items):*   - Amputation - Amputation, closure - Appendectomy - Breast - Burn - Cesarean - Cystectomy - Debridement for necrotizing fasciitis - Dehiscence - Digital gangrene - Disarticulation - Dislocation - Fasciotomy - Herniorrhaphy - Hydrocele - Hysterectomy - Hematoma muscular - Incision and drainage, abscess - Laceration - Laparotomy, acute abdomen - Laparotomy, ectopic pregnancy - Laparotomy, intestinal obstruction - Laparotomy, pelvic peritonitis - Laparotomy, peritonitis - Laparotomy, uterine hemorrhage - Ovarian cystectomy - Preputioplasty - Removal of foreign body - Tumor - Umbilical hernia - Other   Total surgeries by surgical procedure, unspecified urgency *(11 items):*   - Achilles tenotomy - Arthrotomy and joint lavage - Closed reduction, dislocation - Hemipatellectomy - Neurorrhaphy - Osteotomy - Reduction and osteosynthesis, long bone fracture - Reduction under anesthesia - Sequestrectomy - Surgical treatment of open fracture - Tenorrhaphy | Total inpatient surgeries by surgical procedure *(8 items):*   - Cesarean, elective - Cesarean, emergent - Hysterectomy - Inguinal herniorrhaphy, elective - Inguinal herniorrhaphy, emergent - Laparotomy - Tubal ligation - Others   Total surgical discharges | Direct catchment population  Total major surgical  operations  Total surgeries by type  *(variable items)*  Total cesareans  Total surgical admissions  Total surgical admissions by  indication *(variable items)*  Total surgical discharges |
|  | *Mortality* |  | Total inpatient surgical deaths  Total inpatient deaths by surgical procedure *(8 items):*   - Cesarean, elective - Cesarean, emergent - Hysterectomy - Inguinal herniorrhaphy, elective - Inguinal herniorrhaphy, emergent - Laparotomy - Tubal ligation - Others | Total inpatient deaths due to surgery |
|  | *Workforce* |  |  | Total surgeons  Total non-physician surgeons  Total surgical technicians  Total anesthesiologists  Total non-physician  anesthesiologists  Total anesthesia technicians  Total obstetricians |
|  | *Infrastructure* |  |  | Total beds on surgical ward  Total operating tables  Total anesthesia machines  Total functional operating  rooms |
|  | *Quality improvement* |  |  | Total inpatient deaths on  surgical service discussed  during morbidity and  mortality-equivalent  conference  Total avoidable inpatient  deaths on surgical service  Total unavoidable inpatient  deaths on surgical service |
| **José Macamo General Hospital** | **Data Category** | **Variables Collected** | | |
|  | *Volume* | Total surgical admissions  Total surgical discharges by diagnosis *(23 items):*   - Abscess, cervical - Abscess - Acute abdomen - Appendicitis - Burn - Cellulitis - Diabetic foot - Ectopic testicle - Erysipelas - Gangrene - Giant lipoma - Hemorrhoid - Hernia, inguinal - Hernia, scrotal - Hernia, umbilical - Hernia, white line - Hip fracture - Hydrocele - Lipoblastoma - Polytrauma - Renal insufficiency - Skin graft - Varicocele | Total inpatient surgeries by surgical procedure *(8 items):*   - Cesarean, elective - Cesarean, emergent - Hysterectomy - Inguinal herniorrhaphy, elective - Inguinal herniorrhaphy, emergent - Laparotomy - Tubal ligation - Others   Total surgical discharges | Direct catchment population  Total major surgical operations  Number of surgeries by  procedure *(variable items)*  Total cesareans  Total surgical admissions  Total surgical admissions by  indication *(variable items)*  Total surgical discharges |
|  | *Mortality* | Total inpatient surgical deaths | Total inpatient surgical deaths  Total inpatient deaths by surgical procedure *(8 items):*   - Cesarean, elective - Cesarean, emergent - Hysterectomy - Inguinal herniorrhaphy, elective - Inguinal herniorrhaphy, emergent - Laparotomy - Tubal ligation - Others | Total inpatient deaths due to surgery |
|  | *Workforce* |  |  | Total surgeons  Total non-physician surgeons  Total surgical technicians  Total anesthesiologists  Total non-physician  anesthesiologists  Total anesthesia technicians  Total obstetricians |
|  | *Infrastructure* |  |  | Total beds on surgical ward  Total operating tables  Total anesthesia machines  Total functional operating  rooms |
|  | *Quality improvement* | Total cancelled surgeries  Total readmissions to surgical  ward < 72h |  | Total inpatient deaths on  surgical service discussed  during morbidity and  mortality-equivalent  conference  Total avoidable inpatient  deaths on surgical service  Total unavoidable inpatient  deaths on surgical service |
| **Manhiça District Hospital** | **Data Category** | **Variables Collected** | | |
|  | *Volume* | Total surgical admissions by diagnosis *(7 items):*   - Burn - Hydrocele - Inguinal hernia - Infected wound - Lipoma - Pyomyositis - Others   Disposition by surgical diagnosis *(for above 7 items):*   - Transferred - Abandoned - Discharged | Total inpatient surgeries by surgical procedure *(8 items):*   - Cesarean, elective - Cesarean, emergent - Hysterectomy - Inguinal herniorrhaphy, elective - Inguinal herniorrhaphy, emergent - Laparotomy - Tubal ligation - Others   Total surgical discharges | Direct catchment population  Total major surgical operations  Number of surgeries by  diagnosis *(4 items)*:   - Interpersonal violence - Motor vehicle accident - Bites - Work-related injury   Total cesareans  Total surgical admissions  Total surgical admissions by procedure type *(3 items)*:   - Elective herniorrhaphy - Emergent herniorrhaphy - Hydrocelectomy   Total surgical discharges |
|  | *Mortality* | Total inpatient deaths by diagnosis *(7 items):*   - Burn - Hydrocele - Inguinal hernia - Infected wound - Lipoma - Pyomyositis - Others | Total inpatient surgical deaths  Total inpatient deaths by surgical procedure *(8 items):*   - Cesarean, elective - Cesarean, emergent - Hysterectomy - Inguinal herniorrhaphy, elective - Inguinal herniorrhaphy, emergent - Laparotomy - Tubal ligation - Others | Total inpatient deaths due to surgery |
|  | *Workforce* |  |  | Total surgeons  Total non-physician surgeons  Total surgical technicians  Total anesthesiologists  Total non-physician  anesthesiologists  Total anesthesia technicians  Total obstetricians |
|  | *Infrastructure* |  |  | Total beds on surgical ward  Total operating tables  Total anesthesia machines  Total functional operating  rooms |
|  | *Quality improvement* |  |  | Total inpatient deaths on  surgical service discussed  during morbidity and  mortality-equivalent  conference  Total avoidable inpatient  deaths on surgical service  Total unavoidable inpatient  deaths on surgical service |
| **Xinvane Rural Hospital** | **Data Category** | **Variables Collected** | | |
|  | *Volume* | Total surgeries by procedure type *(12 items):*   - Cesarean, elective - Cesarean, urgent - Circumcision - Cystectomy - Hemorrhoidectomy - Herniorrhaphy, elective - Herniorrhaphy, urgent - Hydrocelectomy - Hysterectomy - Laparotomy (for fibroid, cyst and ruptured ectopic pregnancy) - Lipoma - Tubal ligation   Total surgeries by diagnosis *(12 items):*   - Abscess drainage - Animal bite - Assault - Burn - Car accident - Disinfection and dressings - Foreign body - Immobilization with plaster - Sutures - Urinary catheterization, bladder lavage - Work accident - Other | Total inpatient surgeries by surgical procedure *(8 items):*   - Cesarean, elective - Cesarean, emergent - Hysterectomy - Inguinal herniorrhaphy, elective - Inguinal herniorrhaphy, emergent - Laparotomy - Tubal ligation - Others   Total surgical discharges | Direct catchment population  Total major surgical operations  Total surgeries by procedure type *(6 items)*:   - Herniorrhaphy - Lipoma - Ovarian cyst - Myomectomy - Cesarean - Other   Total cesareans |
|  | *Mortality* |  | Total inpatient surgical deaths  Total inpatient deaths by surgical procedure *(8 items):*   - Cesarean, elective - Cesarean, emergent - Hysterectomy - Inguinal herniorrhaphy, elective - Inguinal herniorrhaphy, emergent - Laparotomy - Tubal ligation - Others | Total inpatient surgical deaths  Total inpatient deaths by surgical procedure *(7 items)*:   - Herniorrhaphy - Lipoma - Ovarian cyst - Cystectomy - Myomectomy - Cesarean - Other |
|  | *Workforce* |  |  | Total surgeons  Total surgical technicians  Total anesthesiologists  Total anesthesia technicians  Total obstetricians |
|  | *Infrastructure* |  |  | Total beds on surgical ward  Total operating tables  Total anesthesia machines  Total functional operating  rooms  Equipment availability (gloves,  syringe, blades, suture, other) |
|  | *Quality improvement* |  |  |  |

Abbreviations: DHIS2 – District Health Information Software 2, an open-source platform for health management data (<https://dhis2.org/about-2/#:~:text=DHIS2%20is%20an%20open%20source,management%20information%20system%20(HMIS)>.)
